# An overview of deep learning applications in precocious puberty and thyroid dysfunction

**DOI:** 10.3389/fendo.2022.959546

**Published:** 2022-10-20

**Authors:** Misbah Razzaq, Frédérique Clément, Romain Yvinec

**Affiliations:** ^1^ PRC, INRAE, CNRS, Université de Tours, Nouzilly, France; ^2^ Université Paris-Saclay, Inria, Centre Inria de Saclay, Palaiseau, France

**Keywords:** deep learning, endocrinology, thyroid dysfunction, artificial intelligence, precocious puberty, supervised learning, imbalanced data

## Abstract

In the last decade, deep learning methods have garnered a great deal of attention in endocrinology research. In this article, we provide a summary of current deep learning applications in endocrine disorders caused by either precocious onset of adult hormone or abnormal amount of hormone production. To give access to the broader audience, we start with a gentle introduction to deep learning and its most commonly used architectures, and then we focus on the research trends of deep learning applications in thyroid dysfunction classification and precocious puberty diagnosis. We highlight the strengths and weaknesses of various approaches and discuss potential solutions to different challenges. We also go through the practical considerations useful for choosing (and building) the deep learning model, as well as for understanding the thought process behind different decisions made by these models. Finally, we give concluding remarks and future directions.

## Highlights

- We provide comprehensive cues and synoptic tables to analyze and compare different deep learning-based studies dedicated to endocrinological issues.
- Our critical analysis embraces many criteria related to the dataset building and preprocessing, management of imbalanced data or missing values, selection and implementation of neural network architecture, and use of metrics to assess accuracy and computing results.
- We conclude that:

- Deep learning methods have been applied successfully to clinical endocrinology.
- Deep learning is effective in assessing the biological bone age for precocious puberty diagnosis.
- Deep learning is effective in predicting the thyroid status from standard lab tests.
- We expect that the next generation of deep learning approaches in endocrinology will be improved by including multi-source information.

## 1 Introduction

**Figure 1:**
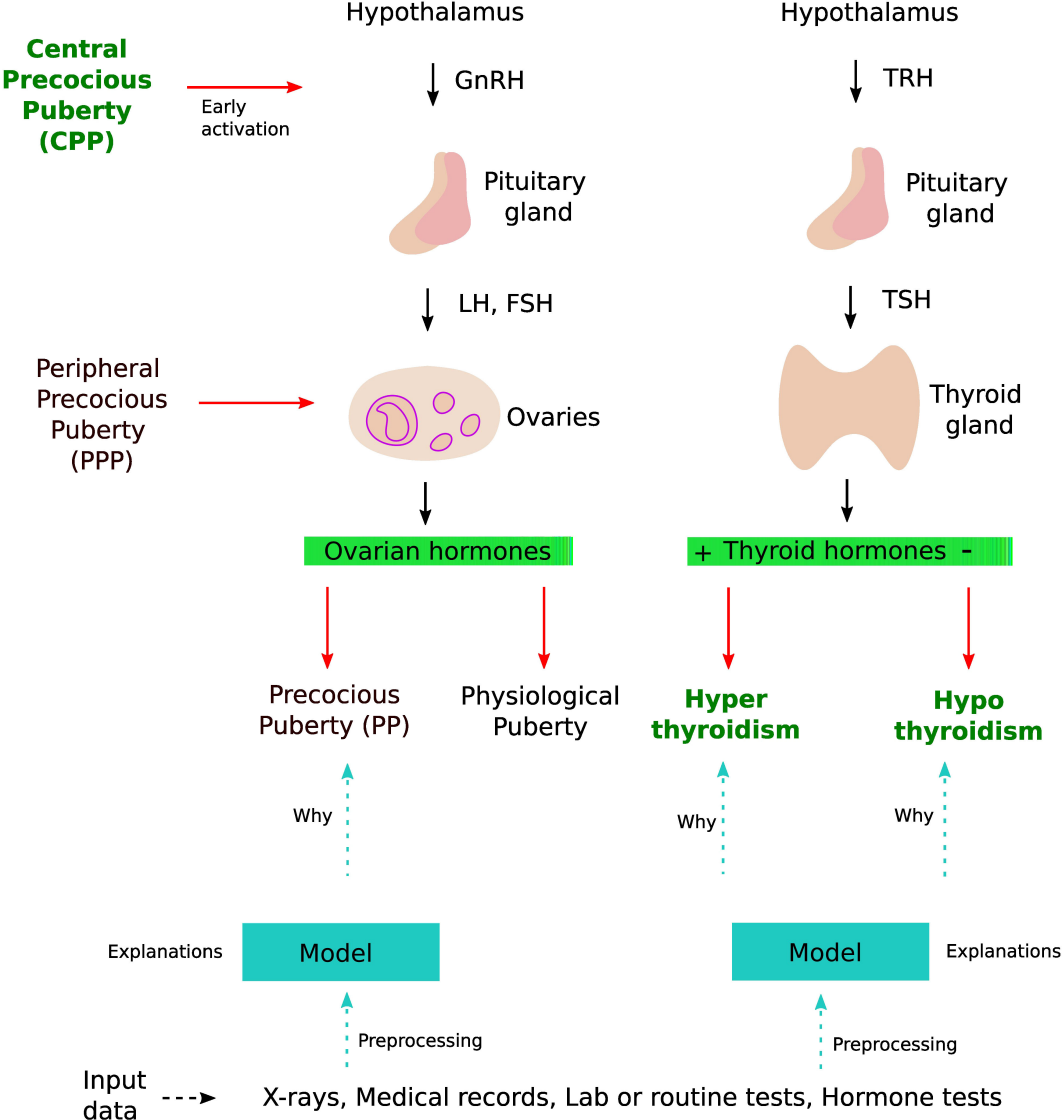
DL application in thyroid dysfunction and precocious puberty. The process starts with building a model from different data sources such as x-rays, medical records, and lab tests. Preprocessing is performed on the dataset to handle issues highlighted in *section 4* (Considerations). To understand the internal mechanism of the DL model, one can use different explanation methods as discussed in *section 4.5* (The black box nature).

In this review, we give an overview of deep learning (DL^1^) methods and their application to thyroid dysfunction and precocious puberty from a diagnostic point of view (see **Figure 1**). Recently, DL methods involving artificial neural networks (ANNs) with multiple layers have become popular to perform classification and regression tasks involving large amounts of data (1). They have been successfully applied in many domains such as image recognition (2), robotics (3), speech recognition (4), and life sciences (5–7). ANNs can deal with complex and noisy data. The layer-wise design of nonlinear processing units enables them to model nonlinear relationships. Advances in biomedical technologies provide us with large amounts of data such as proteomics, genomics, and medical images (8). ANN-based approaches can take raw features (such as images or gene expression profiles) from large datasets as input to create models identifying hidden patterns in the data. These models can then be used to perform predictions on additional datasets. ANNs have shown great results in identifying patterns existing in complex biological data (9).

ANNs are computing systems based on the idea of replicating human brains. An ANN is composed of different units or artificial neurons inspired by the functionality of biological neurons. ANNs map an input space to an output space analogous to a mathematical function. ANNs are quite resilient to noise in datasets as well as in the learning procedure (10). ANNs can be used to perform supervised as well as unsupervised learning (11). In supervised learning, we are given a dataset of (input, output) pairs and our goal is to learn the relationship (function) between these pairs, so that we can predict values for unseen data (12). In a biological context, input can be genomic sequences, gene or protein expression profiles, metabolite concentrations, etc. The output can be growth rates, diseased or healthy states, and sub-types of a disease (13). For example, it can be used to classify patients into two categories of thyroid function, i.e., normal and hypothyroidism, using a proteomics dataset. In such a case, our inputs are the protein levels (continuous variables) and our output is the type of thyroid function (binary variable). Typically, we divide our dataset into three subsets, i.e., training (80%), validation (10%), and testing (10%). ANN is trained using the training set while fine-tuning the parameters using the validation set, and finally predictions are performed on the testing set (12). On the other hand, in unsupervised learning, we have unlabeled data. The objective is to discover useful properties regarding the structure in the data. Various tasks can be performed in an unsupervised manner such as clustering, dimensionality reduction, association learning, and outlier detection. For example, we may reveal groups of proteins whose level of expression is consistent with the above thyroid function classification, which may further lead to the search of biomarkers or signaling pathways responsible for hypothyroidism.

Various machine learning algorithms have been shown to be useful in the diagnosis of endocrine disorders (14). The scope of our study is narrowed to a review of DL methods, which is a sub-field of machine learning using biologically inspired ANNs. There are several reasons why DL can be a useful technique for thyroid dysfunction and precocious puberty diagnosis. In case of precocious puberty, usually, the biological bone age is more advanced than the chronological age (15), and is traditionally measured by Tanner–Whitehouse (TW) (16) and Greulich and Pyle (GP) (17) methods, which take time and supply subjective estimates. Standard machine learning algorithms such as support vector regressions or the gradient boosted decision trees (GBDT) have also been employed for bone age assessment (BAA); however, they require manual feature extraction. On the contrary, DL methods present an ideal framework for BAA. In particular, convolutional neural networks (CNNs) can be used to determine objective bone age estimates using left-hand images. Using CNNs can save the time of radiologists and help identify new features from images related to the biological age. In addition, left-hand radiographs can be merged with other sources of information such as pelvic ultrasonographs (USs) and electronic health records (EHRs) to improve diagnosis. In case of multi-source information, most of the current work use DL methods as a feature extractor and then employ standard machine learning methods to predict bone age. In the future, fully DL methods will probably be developed to handle multi-source information. The diagnosis of thyroid dysfunction, in particular hypothyroidism, is a challenging task, especially since most symptoms are poorly specific. DL methods seem promising to predict thyroid dysfunction from routine clinical tests features, with a high accuracy (18), yet multi-source information are rarely handled so far. This review has been motivated by recent successful applications of DL methods to endocrine issues. We intend to draw the reader’s attention on both the current interest and limits of such approaches, and expected future development. To do so, we first introduce background notions on common DL methods, then we describe in more details the DL approaches dedicated to the diagnosis of precocious puberty and thyroid status. Meanwhile, we provide good practice counseling to help non-experts interpret the results of DL-based studies, preprocess their datasets adequately, and possibly start setting up their own design. For a detailed description of machine learning techniques for thyroid disease, the interested reader can consult the following review (19). An overview on bone age assessment in different contexts including precocious puberty using traditional and machine learning methods can be found elsewhere (20, 21).

In section 2, we start with an easy-to-understand overview of different DL architectures, i.e., multilayer perceptrons (MLPs), CNNs, self-organizing map (SOM)-based neural networks, and Bayesian regularized neural networks (BRNNs). Then, we move toward the DL application in diagnosing precocious puberty (section 3.1), where mostly CNN-based architectures have been employed. We start with describing the background of the problem. Then, we argue why precocious puberty is an ideal domain for the application of DL methods. We highlight common concerns such as dealing with data heterogeneity and the black box nature of DL models, and discuss how they can be addressed. Subsequently, we discuss thyroid dysfunction classification in section 3.2 as a second case study for applying both supervised (using MLPs and BRNNs) and unsupervised (using SOMs) learning-based DL methods. We also highlight how the combination of supervised and unsupervised learning helps to interpret or explain the decision boundaries of DL models. We discuss the power and weakness of different DL models in this specific application. Finally, we discuss in section 4 the common issues that one should consider when applying DL methods.

## 2 Artificial neural networks

**Figure 2:**
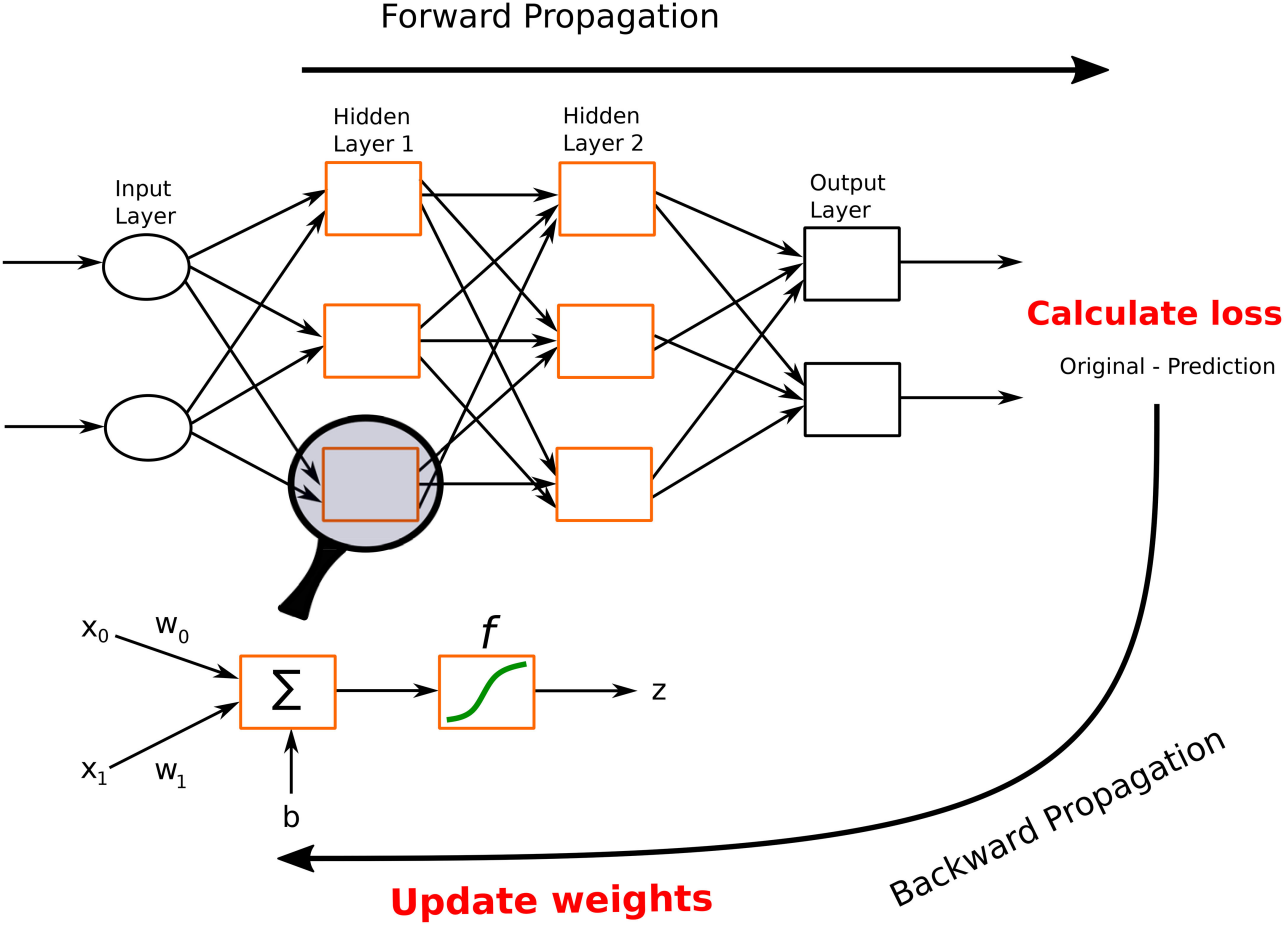
A feed-forward neural network. This network consists of one input layer, two hidden layers, and one output layer. The input nodes (shown as ovals) forward their inputs without performing any transformation. The nodes (shown in orange rectangles) in the second (hidden) layer perform more complex and abstract tasks, for example, learning nonlinear decision boundaries. The output layer contains two nodes (shown in black rectangles). The functionality of one of the hidden neurons is highlighted at the bottom of the figure. It is worth noting that neurons are connected across layers, but not within layers.

In this section, we describe different architectures of ANNs that are subsequently used in the different DL applications in the thyroid dysfunction classification and precocious puberty diagnosis. Different ANN architectures (see **Table 1**) are generally better suited for specific types of tasks (for example, CCNs perform well for image classification or object detection). Yet, the core process behind most architectures is similar. Neural networks perform classification or regression tasks by learning a function between inputs and outputs through training. Neural network training imply two main phases (see **Figure 2**): (i) forward propagation and (ii) backward propagation. In the forward propagation, outputs of all nodes while moving from the input layer to the output layer are generated. At the output layer, error between the predicted output and the expected output is computed. In the second phase, the error is backpropagated to update the network parameters. These phases are iterated so as to minimize the final error by adjusting the values of connections between nodes. Once learning is achieved, the DL network is run on the testing dataset, and several criteria are used to assess the accuracy of the predictions (see **Table 2**).

**Table 1:** The neural network architectures.

Type	Architecture	Learning rule
Supervised Unsupervised	Multilayer perceptrons (MLPs) Convolutional neural networks (CNNs) Bayesian regularized neural networks (BRNNs) Self-organizing maps (SOMs)	Error correction Error correction Error correction Competitive

**Table 2:** Different performance metrics for classification tasks.

Metric	Formula	Description
Accuracy	$\frac{Correction\;predictions}{All\;Predictions}$	Proportion of correct predictions among all predictions
Root mean square error (RMSE)	$\sqrt{\frac{\sum_{i=0}^{m}{\left(o_{i}-y_{i}\right)}^{2}}{m+1}}$	Square root of the difference between the model predictions and actual values
Mean absolute difference (MAD)	$\frac{\sum_{i=0}^{m}/o_{i}-y_{i/}}{m+1}$	Absolute difference between the model predictions and actual values
True positive rate (TPR)	$\frac{TP}{TP+FN}$	Proportion of correct positive predictions among all positive cases
False positive rate (FPR)	$\frac{FP}{FP+TN}$	Proportion of incorrect positive predictions among all negative cases
False negative rate (FNR)	$\frac{FN}{FN+TP}$	Proportion of incorrect negative predictions among all positive cases
True negative rate (TNR)	$\frac{TN}{TN+FP}$	Proportion of correct negative predictions among all negative cases.

Here, o represents the actual values and y represents the predicted values. FP is the number of false positives. TP is the number of true positives. TN is the number of true negatives. FN is the number of false negatives.

### 2.1 Multilayer perceptrons

MLPs, also called feed-forward neural networks (FFNNs), are the simplest architecture of neural networks. Information are conveyed unidirectionally from the input to the output layer, through the hidden layers (see **Figure 2**). More elaborate networks, called recurrent neural networks (RNNs) (22), include feedback loops between the network layers.

Formally, we can define the functionality of an artificial neuron in the following manner:


(1) $$z=f\left(\sum_{i=0}^{n}w_{i}x_{i}+b\right)$$

**Figure 3:**
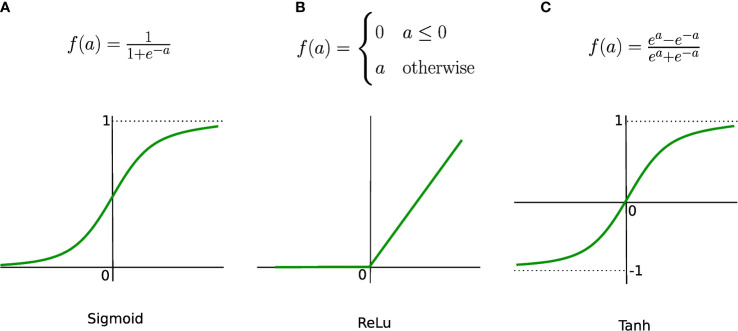
Most common activation functions. **(A)** Sigmoind, **(B)** ReLu and **(C)** Tanh.

Where *x*_0_,*x*_1_, … *x_n_* are the inputs, *w*_0_,*w*_1_, … *w_n_* are the weights associated with the respective inputs, *b* represents the bias, *z* is the output of the neuron, and *f* is an activation function. When converting an input signal into an output signal, activation functions (also known as transfer functions) are crucial. Activation functions can be formulated as thresholds on the current inputs, above which neurons are activated. In order to learn complex nonlinear relations, a nonlinear activation function is unavoidable. The hyperbolic tangent (Tanh), logistic sigmoid (also called sigmoid), and rectified linear unit (ReLu) functions are the most popular activation functions (see **Figure 3**). The graphs of the sigmoid and Tanh functions form an S-shaped curve with output values bounded by (0,1) and (-1,1), respectively. Within the last few years, ReLu has become the most popular activation function. ReLu returns zero for any negative input value, and the input value otherwise, so that outputs are not bounded (lim_a_→+∞ f(a) = +∞).

**Figure 4:**
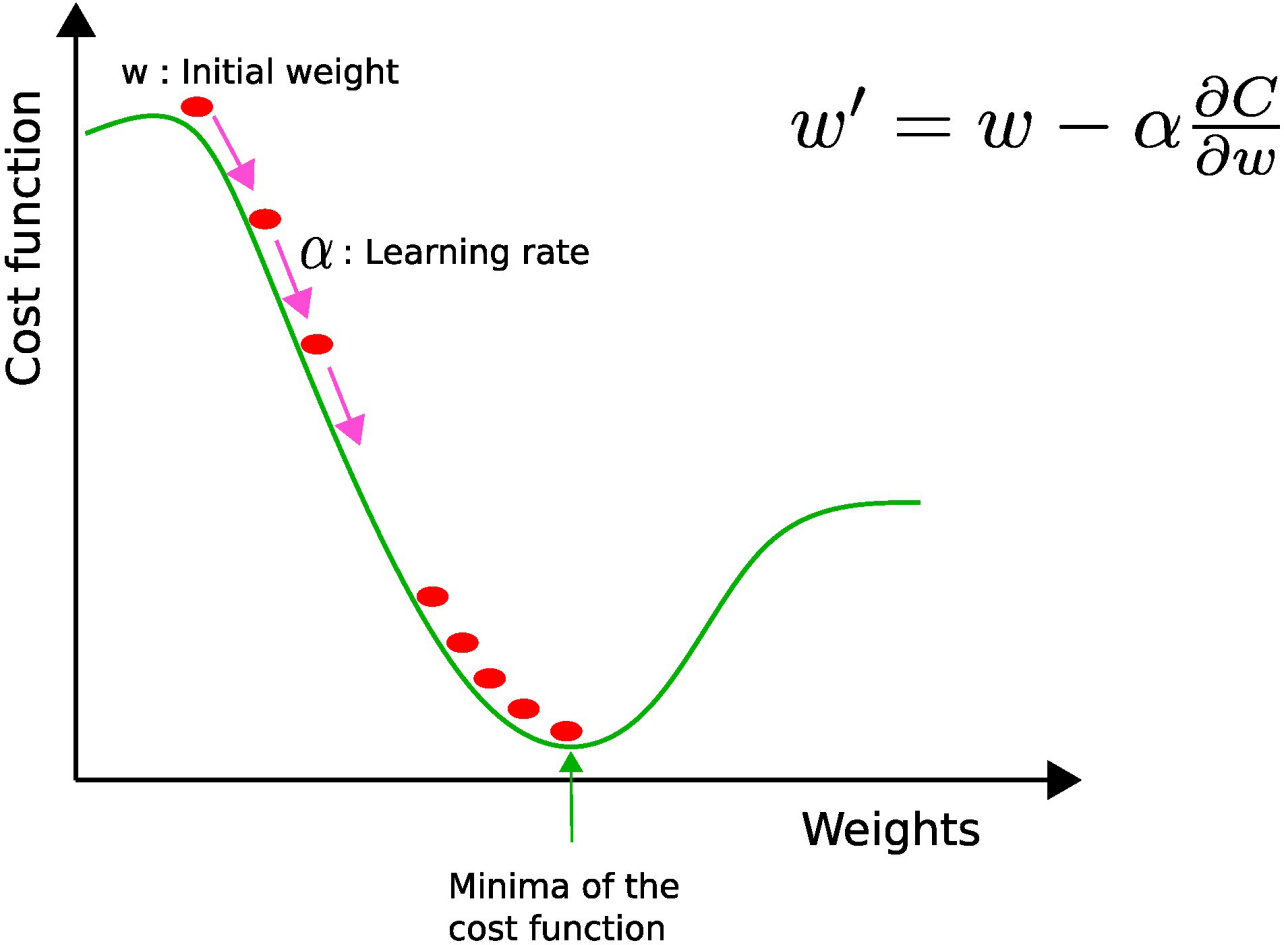
Gradient descent: w and w′ represent the current and updated weights, respectively, and *α* is the learning rate. The current weight w is moved along the direction of the steepest descent (pink arrow) by the learning rate *α*.

After determining the number of layers, nodes per layer, and activation functions, the neural network can be trained. To determine the error between expected and predicted outputs, one chooses a cost function. The quadratic (mean square) cost function can be defined as:


(2) $$C=\frac{1}{2(m+1)}\sum_{k=0}^{m}{\left(o_{k}-y_{k}\right)}^{2}$$

where vector *y* = (*y*_0_,…,*y_m_*) contains the predicted outputs, vector *o* = (*o*_0_,…,*o_m_*) contains the expected (“right”) outputs, and *m*+1 is the size of the training samples. The cross-entropy cost function is used in case of binary outputs, for instance in classification problems, and is defined as:


(3) $$C=-\frac{1}{m+1}\sum_{k=0}^{m}\left[o_{k}ln\;y_{k}+(1-o_{k})ln(1-y_{k})\right]$$

where the *o_k_*’s are the expected labels, i.e., 0 or 1, and the *y_k_*’s are the continuous-valued predictions of the model. The next step is to learn the optimal values of weights and biases by minimizing the cost function. The most popular algorithm to perform parameter optimization for neural networks is the gradient descent (see **Figure 4**). This iterative algorithm tries to find the local minima of the cost function by performing a first-order partial differentiation with respect to learnable parameters. During each iteration, the parameter values are updated when the error is backpropagated. This process is repeated until the function has decreased below a fixed threshold or the maximal iteration number is reached.

The value of the learning rate (*α*) determines the size of each step toward the local minimum. If the learning rate is too high, oscillations may occur, while if it is too low, the computational cost needed to converge becomes penalizing. Therefore, it is better to use an adaptive learning rate, i.e., a bigger learning rate in the beginning to reduce computational overhead and a smaller one toward the end in order to fine-tune the parameters (8).

There exist several variants of the gradient descent algorithm. The batch gradient descent calculates the error for all examples (pairs of input and output) of the training set. All parameters are updated exactly once in one iteration, which is extremely memory intensive because data must be stored in memory. The stochastic gradient descent update the parameters *m*+1 times. Each time, it calculates the error for a single random sample of examples, which consumes less memory, but can cause the error to fluctuate rather than decrease. The mini-batch gradient descent calculates the error on a subset of the training set. This strategy minimizes the inherent oscillations of stochastic gradient descent while enhancing the efficiency of batch gradient descent.

### 2.2 Convolutional neural network

CNNs are a special type of FFNNs inspired from human vision, and mainly used to perform image classification, object detection, and clustering similar images. They are based on three layer types (23): convolutional layers for feature extraction, pooling layers for dimensionality reduction, and fully connected layers for classification.

**Figure 5:**
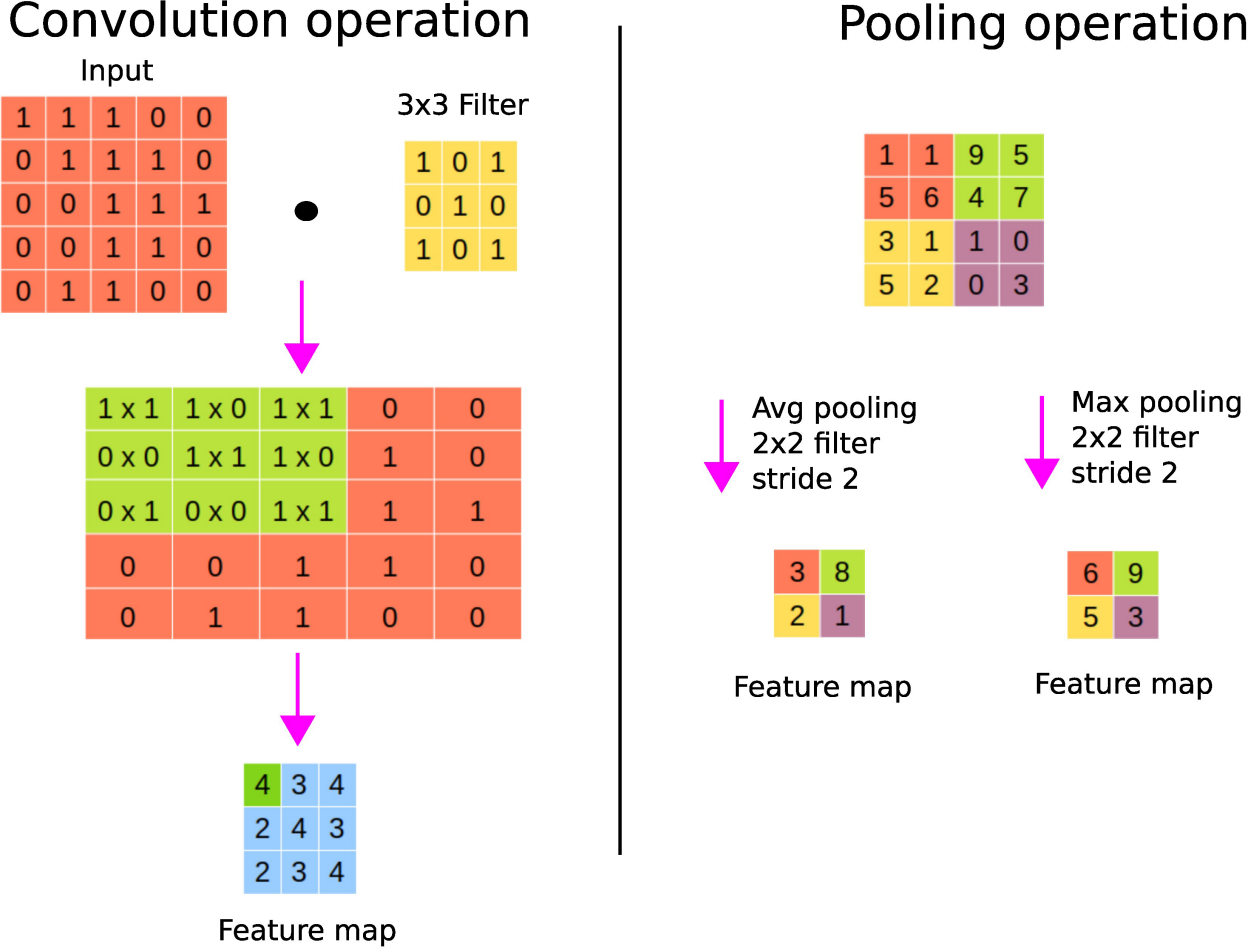
CNN’s fundamental operations. On the left panel, a convolution operation is shown where the sum of the element-wise multiplications using a filter across the input data is performed to generate a feature map. On the right panel, average and maximum pooling operations are shown.

A convolutional layer basically first performs element-wise multiplications using different filters or kernels (matrices of numbers) applied to the input data, and then sums the results to generate feature maps. Usually, there are many filters responsible for extracting different types of visual information such as edges, diagonal lines, and orientation from the image, hence generating many feature maps. **Figure 5** shows an instance of the convolution operation with a 3 × 3 filter. These filters are learned during the training process and shared across the input instances. The parameter sharing property of CNNs reduces the storage requirement and guarantees translational equivariance; if we shift the object in the input, then the convolution output will shift equally (22). The output of the convolutional layer is subjected to an activation function such as ReLU to account for nonlinearity. As we can see from **Figure 5**, we may lose border information, which can be avoided by padding, i.e., supplying zeros vertically and horizontally to conserve the edge or border information.

A pooling layer is used to perform the sampling of the feature maps in order to conserve only important information, thereby getting rid of noise and redundancy. Pooling enables CNNs to be invariant to small translations; spatial translation has little effect on the output of the pooling operation (22). For example, a CNN can detect a cat in an image regardless of its position. Max pooling keeps the maximum value of each patch of the feature map, while average pooling keeps the average. **Figure 5** shows the pooling operation with a 2 × 2 filter with stride 2. Stride refers to the step size used for the pooling operation, for example, a stride of size 2 allows to step 4 pixels (2 vertically and 2 horizontally). Large strides allow one to shrink the size of the output. It is worth noting that the parameters (such as filter sizes or operations or strides) of the pooling layer are fixed during the learning process. Fully connected layer(s) are finally used after pooling to perform classification or regression tasks (24).

**Figure 6:**
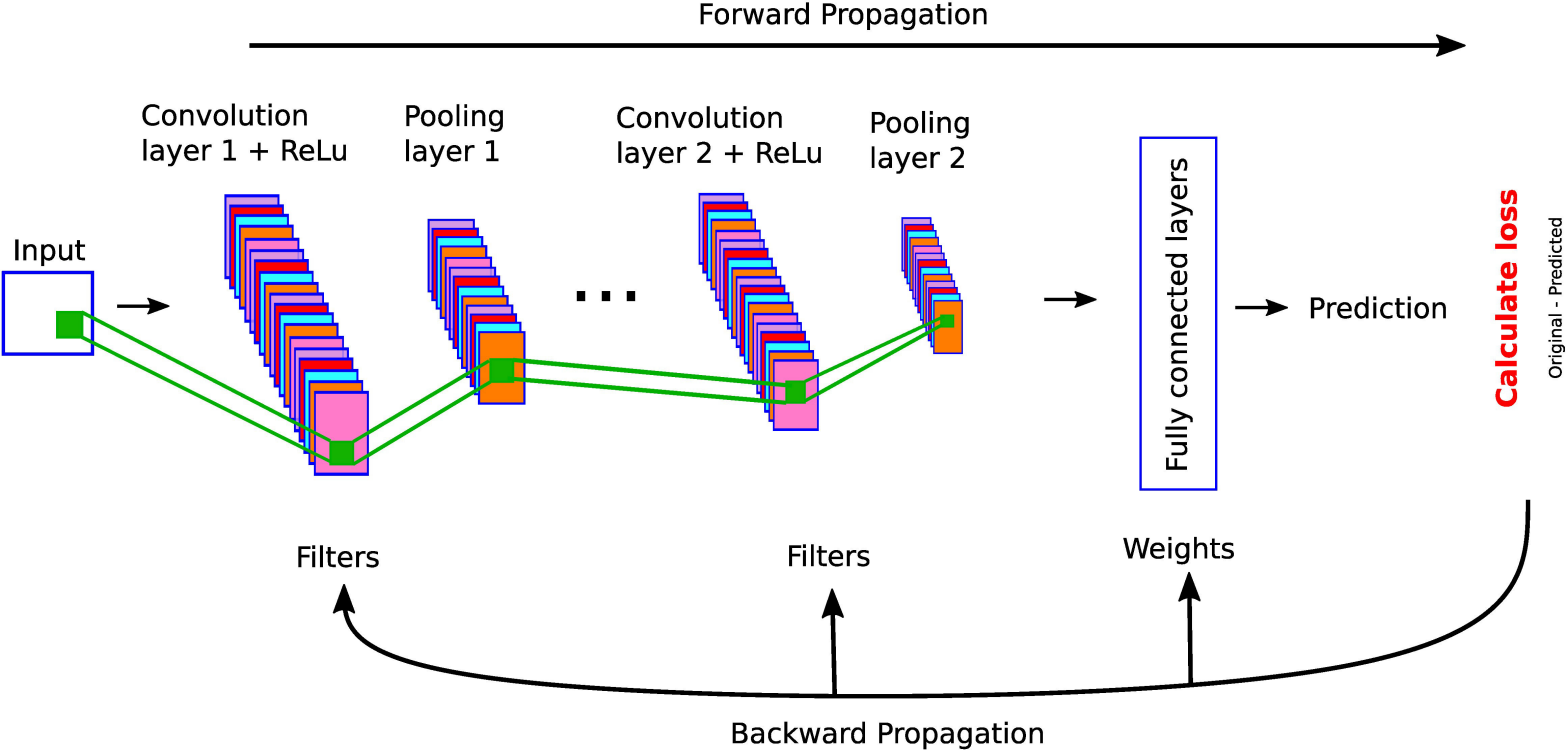
A simple CNN architecture with convolutional, pooling, and fully connected layers.

In **Figure 6**, we show an example of the layer-wise architecture of a CNN.

### 2.3 Self-organizing maps

**Figure 7:**
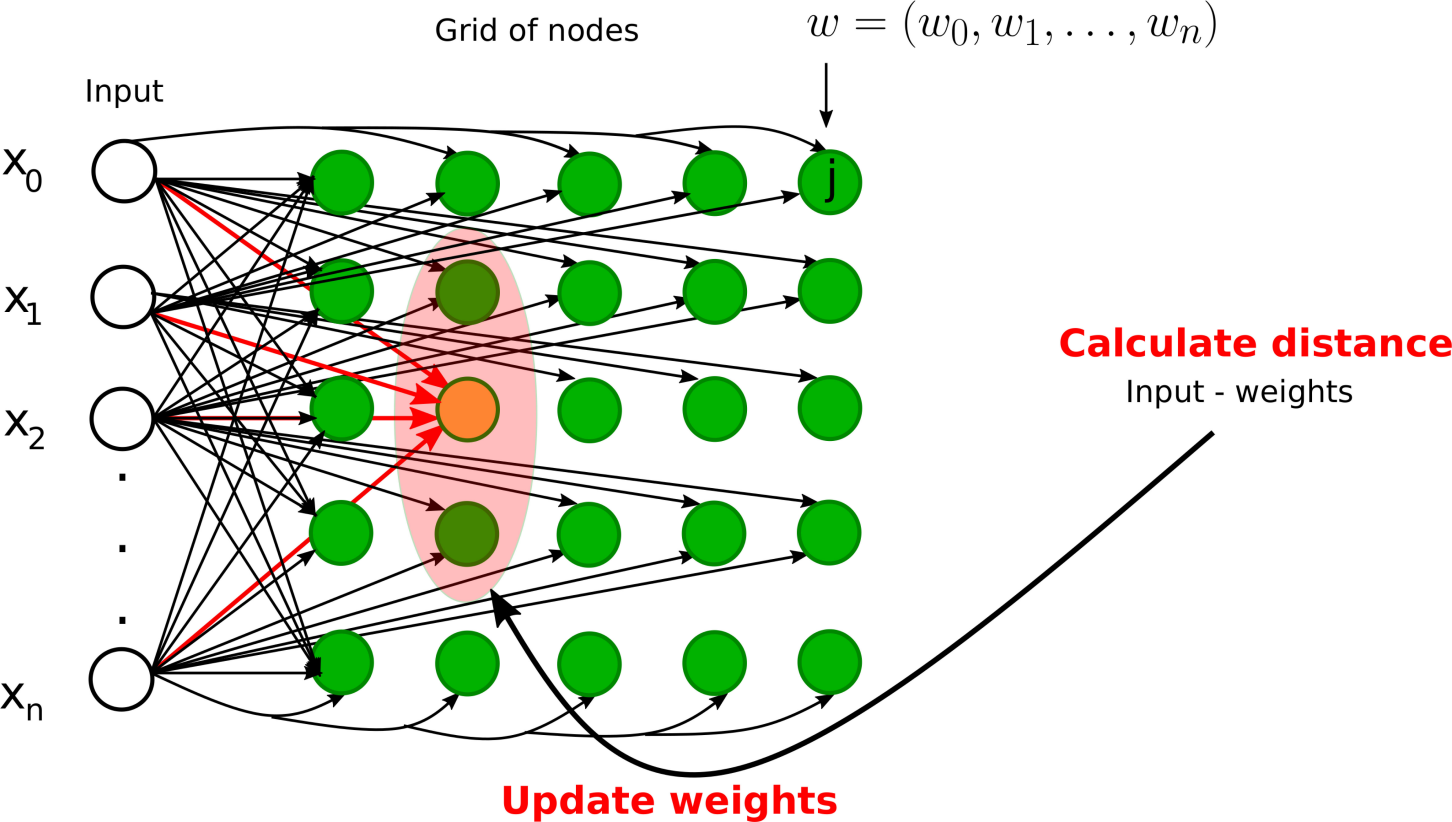
Graphical illustrations of self-organizing neural networks. Instance of a Kohonen neural network with *n* input nodes and a grid of output nodes. Note that each input node is directly connected to a node on the grid. However, we did not draw some of the connections from inputs to the nodes on the grid for clarity. The orange node represents the winner node and the light pink node denotes the neighborhood radius. The weights of the winner node and its neighbors will be updated during the training phase.


The most popular SOMs architecture is the Kohonen network, proposed by Teuvo Kohonen (25, 26) and based on the principle of competitive learning. It is an unsupervised learning method where output neurons compete with each other to become active (11). The output neuron learns to represent different input categories. SOMs are mostly used to classify and visualize high-dimensional data into a lower-dimensional space (typically a 2D space). There are two types of layers in SOM-based neural networks: (1) input layer and (2) output layer (competitive or Kohonen layer), see **Figure 7**. Each node in the input layer is connected to all the nodes in the output layer. Each node in the output layer is characterized by a weight vector whose size is equal to the number of connections to the node. Contrary to other ANNs (see **Table 1**), the weights are adjusted according to learning rules instead of the error computation: either the winner takes all (only the winner weights are updated) or the winner takes most (the weights of both the winner and its neighborhood are updated). The best matching unit or the winner node is identified by calculating the distance between the input and the weight vector. The Euclidean distance is generally used to identify the winner node. We define the Euclidean distance *Dist_j_* between node *j* and input vector *x* of dimension *n*+1 as

(4) $$Dist_{j}=\sum_{i=0}^{n}{\left(x_{i}-w_{i}\right)}^{2}$$

where *w* is the weight vector of node *j* having the same dimension as the input vector. However, other measures such as correlation, direction cosine, and block distance can also be used.

The training process starts with the random initialization of weights. An example is selected from the training set, the winner node is found by calculating the Euclidian distance, the weights of the winner node and/or the neighbor nodes are updated, and this process is repeated until the maximum number of iterations is reached or the modification in weights is less than a predefined threshold. The weights are modified in such a way that the different locations of SOMs reflect distinct categories of the input data. In the testing phase, the weights are fixed and data are projected onto the learned map.

### 2.4 Bayesian regularized neural networks

ANNs are powerful universal approximators that can learn meaningful patterns from extremely complex datasets (27). However, they can also be a victim of overfitting. Overfitting occurs when the model fails to generalize to the test dataset since it approximates too closely the examples from the training dataset; the model has memorized the noise instead of learning the actual signal. During training, overfitting can be detected by verifying the performance on the validation dataset in addition to the training dataset (23). Overfitting can be avoided by regularization to reduce generalization errors (22). The most effective method is to expand the training datasets, which is not always possible. Another option is to use data augmentation, which entails creating the augmented or fake dataset using transformations like rotation and translation. We can also lower the model complexity by reducing the number of layers and nodes.

**Figure 8:**
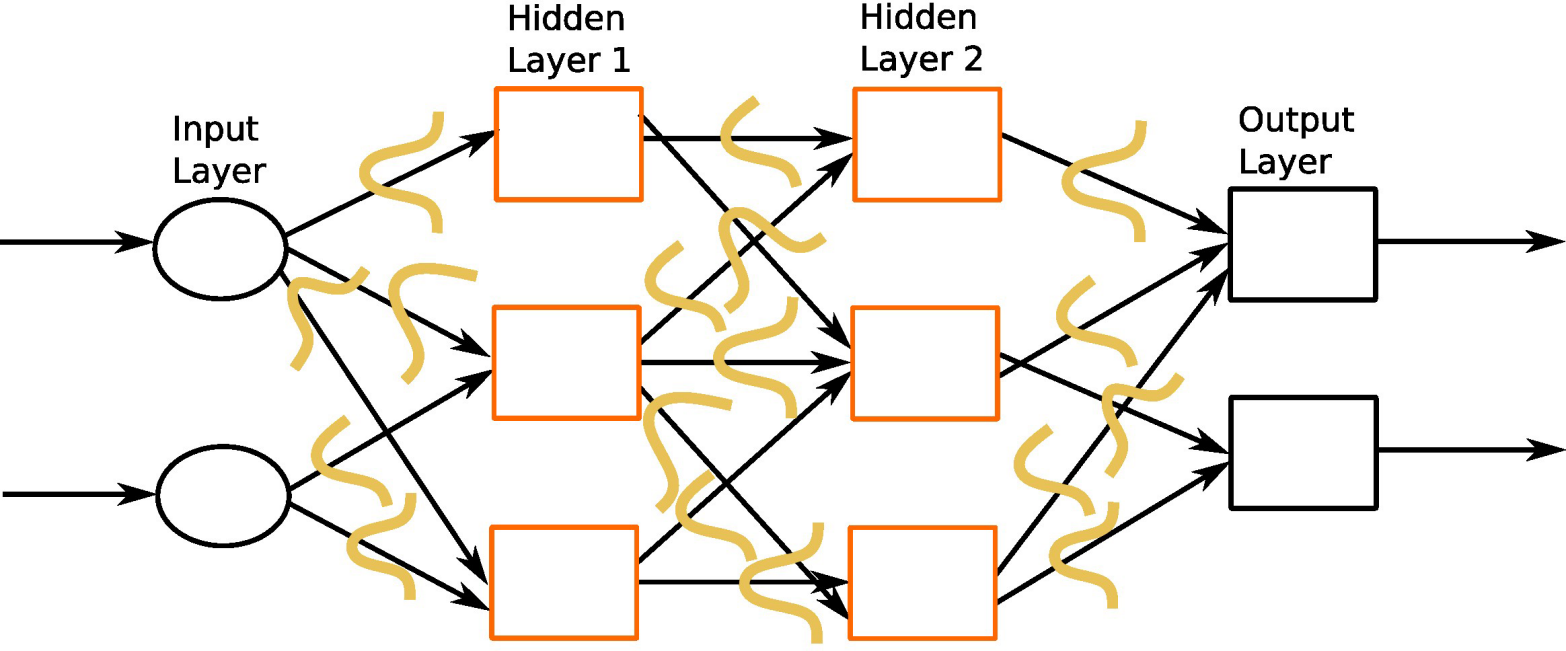
An instance of Bayesian regularized neural network with one input layer, two intermediate hidden layers, and one output layer. The orange curves represent prior distribution on each weight connection.

Bayesian regularized neural networks (BRNNs) are particularly well suited for overcoming the overfitting issue of standard ANNs. In MLPs, a single set of weights are learned through error correction procedure during the training phase, and then these values are used to perform the predictions from the testing dataset. In contrast, in BRNNs, network weights are random variables, whose probability distribution is learned during a training phase (see **Figure 8**), and then weights are drawn from these distributions to make predictions on the testing dataset. The prior distribution of these weights represents the prior belief about the network (prior to the training phase). It is generally difficult to guess what the prior distribution should look like, so that we can use general properties to represent the prior belief, such as smoothness, or use a Gaussian distribution. The posterior distribution is learned for these parameters using Bayesian inference, given the training dataset (28).

In BRNNs, regularization is achieved by introducing a regularization term in the cost function that penalizes large weights resulting from overfitting (equation 5). In the case of centered Gaussian prior distributions, this amounts to using the sum of squares as a penalty term:


(5) $$F=\gamma E_{d}+\beta E_{w},\;E_{w}=\sum_{k=0}^{m}w_{k}^{2}$$

where *E_d_* is the cost function given in equation 2. *β* and *γ* are regularization hyperparameters. The BRNN tries to strike the balance between the prediction error (*E_d_*) and the weights (*E_w_*) by finding the optimal values of parameters *β* and *γ*. The values of *β* and *γ* are not known in advance and learned during training. If *γ* is much larger than *β*, the algorithm favors the goodness of fit, at the expense of keeping a rather high level of model complexity. If *β* is much larger than *γ*, the weight distribution will concentrate around a zero mean (for centered Gaussian prior distributions), meaning that many connections will be removed within the network. In each iteration, the values of both the model parameters (weights) and hyperparameters (*β* and *γ*) are updated. The simplest way is to move the hyperparameter values on a grid from one iteration to the next one. A more elaborate procedure updates the hyperparameter values at the end of each iteration, from the current values of the weights and cost function components *E_w_* and *E_d_* (27).

A great advantage of BRNNs is that they can handle small datasets; the embedded regularization procedure allows one to only divide the original dataset into a learning subset and a testing subset (27). In addition to resolving overfitting, BRNNs propose automatic relevance determination (ARD) (28, 29) to calculate the importance of each variable and ignore less important variables.

## 3 Application of deep learning in endocrinology

### 3.1 Precocious puberty

Puberty is a complex transitional process that is initiated by the activation of the gonadotropic axis and especially by the onset of pulsatile GnRH (gonadotropin-releasing hormone) secretion from hypothalamic neurons. Stimulation of the gonads by the pituitary hormones FSH (follicle-stimulating hormone) and LH (luteinizing hormone) results in steroid synthesis and emergence of secondary sexual characteristics. Precocious puberty (PP) is defined as the development of secondary sexual characteristics before 8 years of age in girls and before 9 years of age in boys (30). PP is further classified into either central precocious puberty (CPP) or peripheral precocious puberty (PPP) (see **Figure 1**). CPP is the most frequent form of PP and is induced by the early activation of the hypothalamic–pituitary–gonadal axis. CPP is usually diagnosed using bone age assessment (BAA), hormonal tests, GnRH stimulation tests (a gold standard), human chorionic gonadotropin levels, and magnetic resonance imaging (31, 32). In a GnRH stimulation test, the response of the pituitary gland is assessed through monitoring FSH and LH blood levels before and after a GnRH hormone shot.

The bone age is typically more advanced than the chronological age in case of PP (33). BAA is an ideal example for CNN application as the goal is to perform the classification of a given set of images. BAA is conventionally assessed from left-hand radiographs, mainly for historical reasons. Since most people are right-handed, the left hand of manual workers was less likely to be impaired (20).

**Figure 9:**
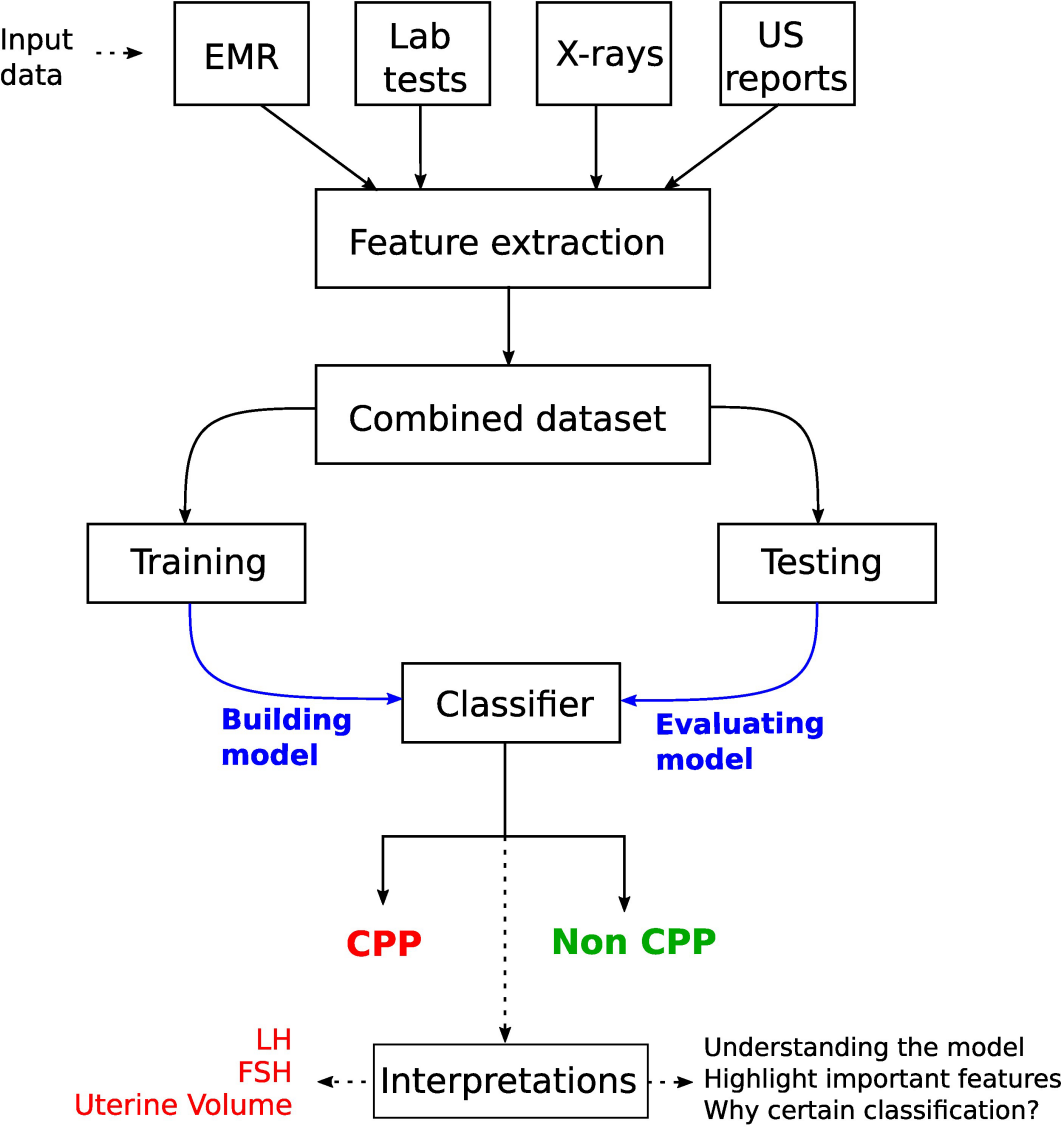
Workflow for a CPP diagnostic system by considering multiple sources of information in young girls. A key step is the interpretation of the model prediction, which highlighted the usefulness of LH and FSH levels (from laboratory tests) and uterine volume (obtained from pelvic ultrasonography).

The TW (16) and Greulich and Pyle (GP) (17) methods are commonly used to assess BAA. GP is the most frequently used method in clinical practice, where radiographs of the left hand and wrist are compared with an atlas of standard bone images to estimate the bone age. The TW method (and its variants) is based on scoring a specific selection of hand and wrist bones, and estimating the bone age by summing these scores. It is more accurate than the GP method (34). In the following, we also discuss studies taking into account multiple sources of information such as ultrasonography (US) reports, health records, and lab tests in addition to radiographs (see **Figure 9**). A synthetic overview on the DL approaches analyzed and compared in the next subsections is provided in **Table 3**.

**Table 3:** DL-based studies for precocious puberty diagnosis.

	Study	Method	Reference	Performance		Time	Source code	Size	Interpretation	Type	Gender
				Accuracy	MAE						
Single Source	(35)	GP	Radiologists	57%, 61%	–	–	No	8,325	Yes	CNN	♀/♂
	(36)	GP	Radiologists	70%	–	29%	No	200	No	CNN	♀/♂
	(37)	GP	Radiologists	–	0.79	–	Yes	1,391	Yes	CNN	♀/♂
	(38)	–	–	91%	–	–	No	301	No	CNN	♀/♂
	(39)	GP	Radiologists	–	–	–	No	1,2585	Yes	CNN	♀/♂
	(40)	TW	Endocrinologist Radiologist	–	0.5 y	–	Yes	9,059	Yes	CNN	♀/♂
	(41)	GP	Radiologist	–	11.1 m	–	Yes	16,810	No	CNN	♀/♂
	(42)	Sauvegrain	Researchers	–	0.22 y	–	No	4,437	Yes	CNN	♀/♂
Hybrid system	(43)	GP + TW	Endocrinologist Radiologist	–	0.39 y	35%	No	15,611	Yes	CNN	♀/♂
	(44)	TW	Radiologist Endocrinologist	–	6.99, 6.99	–	No	14,311	No	FR-CNN + RNN	♀/♂
	(45)	TW	Endocrinologist Radiologist	–	6.07	–	No	12,611	No	ELM + RNN	♀/♂
Multi source	(46)	GP	Radiologist	–	0.61, 0.73	–	No	21,391	No	CNN + SVR	♀/♂
	(47)	GP	Radiologist	–	0.48,0.51	–	No	24,851	No	FR-CNN	♀/♂
	(48)	–	–	–	-	–	No	2523	Yes	DL + XGBoost	♀
	(33)	–	–	68%	–	–	No	2228	Yes	VAE + GBDT	♀

Regarding imaging data, hand images were used except in (42) where elbow images were used as input. The - sign implies that information was either missing or irrelevant for the corresponding study or another evaluation criterion was reported. Two evaluation metrics are reported whenever we find separate scores based on either gender (male or female) or data (public or private).

#### 3.1.1 Bone age assessment

Recently, DL methods have been employed for the BAA, an important parameter in the diagnosis of PP. Lee et al. (35) proposed a fully automated DL system based on CNNs for BAA. Recall that CNNs consist of convolutional and pooling layers, followed by the fully connected layer (see section 2.2). The dataset consists of 4,278 radiographs for women and 4,047 for men. First, they normalized the dataset images to remove discrepancies across images such as different background colors, object colors, and sizes, then performed the BAA using CNN, and finally generated radiology reports. Their system achieved an accuracy of 57.32% for the female cohort and 61.40% for the male cohort. The biggest advantage of the proposed system is that it can take into account images of different formats and qualities in the training step. In addition, they obtained attention maps to visualize image segments used by the model to make a specific decision. Notably, these segments were in accordance with the features employed by experts. Later, a DL system based on the GP method was proposed by Kim et al. (36). They also used a CNN-based DL architecture, and investigated three scenarios: (1) the score decided by the DL system, (2) the score determined by the DL system together with radiologists, and (3) the score produced by the GP technique together with radiologists. Two experienced radiologists created the reference scores. The input dataset consists of left-hand radiographs from 200 patients. The concordance rate between the score proposed by the DL-based system and the reference score was 69.5%, while the reading times of the radiologists decreased by 29% without compromising accuracy. The authors claim to perform better than Lee et al. (35) with an accuracy of 69.5%, which is not fair given the different datasets used for training or testing, and reference values obtained by different experts. Nonetheless, both works provide an excellent example of DL application in the clinical assessment of bone age.

In (37), the authors published a first publicly available DL system “BoNet”, based on a CNN architecture, along with the source code to support the result reproducibility. BoNet was tested on a publicly available dataset of 1,400 images (49) and managed to outperform four previously implemented methods (49–52). In addition, they performed comparison between the features used by the TW method and those used by BoNet for age prediction. While there were some common features used by BoNet and TW methods for BAA, only BoNet highlighted the weak role of carpal bones and, in contrast, the importance of the radius and ulna. This implies that some features now used by clinicians may be unnecessary, while others should be considered.

In (38), the authors have shown the power of data augmentation to deal with scarce medical datasets. They augmented 301 cases of x-rays by 30 times and built a CNN to classify subjects into different age categories. Data augmentation was done by random rotation or brightness regulation of different images. Their CNN model achieved an accuracy of 91.3% on a testing dataset beating some of the existing methods (53). In (39), a multitask CNN model was proposed to estimate bone age as well as to localize ossification centers in different bones, i.e., phalangeal, metacarpal, and carpal (as done in the TW method). They used a dataset from the RSNA Pediatric Bone Age Machine Learning challenge (54), containing 12,585 x-ray hand images. They highlighted the relevant image segments using Gradient-weighted Class Activation Mapping (Grad-CAM) (55), showing that joint learning improved the accuracy of the network by focusing on features, i.e., 20 ossification center regions relevant for bone age assessment.

The authors in (40) proposed a first diagnostic DL system based on an updated version of the TW method (56) with a sample size of 9,059 images. The proposed CNN model achieved a stable overall performance in terms of accuracy and time as compared to the experienced endocrinologists and radiologists. In (41), the authors used the chronological age as a reference (or ground truth) contrary to the reference ages based on GP or TW methods utilized in most of the previous studies. They used 15,129 hand radiographs for training and 1,681 for testing purposes. In addition, they used 214 hand radiographs from an external institute to gauge the generalizability of their CNN model. The suggested DL model performed similarly to GP-based systems or human experts. However, it was more sensitive to systematic biases such as overestimating the age of younger children. In order to deal with the limitations of hand x-rays such as large inter-observer error and subtle morphological changes in hand or wrist bones, the authors in (42) presented the first DL model for BAA using elbow radiographs. They obtained 4,437 images to train and validate a CNN model from one institute. A set of 141 images were obtained from an external hospital for testing purposes. Their model yielded results that were equivalent to those of human experts.

#### 3.1.2 A hybrid system

Recently, Lee et al. (43) suggested a hybrid GP- and TW-based DL system. Two public datasets were used to train a CNN model: (1) 14,236 radiographs from RSNA (54) and (2) 1,375 radiographs from Digital (57). The reference scores were generated by three experts (endocrinologists and radiologists). Their technique first computes two types of BBA using CNN based on the TW and GP methodologies, and then integrates the features from both the TW and GP methods to propose a final BAA using a fully connected neural network. There was an excellent agreement between the reference BAA and the predicted BAA with a mean absolute difference (MAD) of 0.39 years (95% confidence interval, 0.33-0.45 years) and reading times were reduced by 35% with the help of the DL system.

In (44), a TW-based DL system combines an RNN (with a modified optimization process) as a classifier with an FR-CNN (Faster Region-CNN) as a feature extractor. This hybrid model was tested using public and private datasets, with better results than models based on single architecture and non-modified optimization process. To decrease the computational overhead, a TW hybrid system based on DL and extreme machine learning (ELM) was proposed in (45). ELM is a single hidden layer FFNN, introduced in (58). It assigns fixed values to the weights linking the input and hidden nodes, so that only the weights linking the hidden and output nodes are tuned, which makes it extremely faster (59). Like the TW method, only selected parts of the images were used for the BAA, thereby reducing the computational cost. Feature extraction was performed using a CNN, and retrieved features were then used as inputs to the ELM algorithm to predict bone age. They used a publicly available dataset of 12,611 images, which was divided into a training (70%) and a testing (30%) set. Their trials have revealed that the CNN-based model outperforms the others, although it necessitates more computational resources. In contrast, their hybrid system is slightly less accurate than the CNN-based system, but it is computationally more economical.

#### 3.1.3 Multiple sources of information

Multiple sources of information can help to improve diagnoses in the medical field (60, 61). In **Figure 9**, we illustrate a generic workflow to perform CPP diagnosis with a DL model using diverse sources of information.

In (46), the authors have taken into account x-ray images in addition to race and gender (male or female) information. They model the problem of age prediction as a regression task whose ultimate goal is to predict the age as close as possible to the ground truth. They used a CNN as a feature extractor in x-ray images, and combined the output features with the race and sex to get the bone age estimation using support vector regression. They used both public and private datasets to construct and test the models. Their results show that merging heterogeneous features can improve bone age estimates of the model. Later, in (47), the authors adopted FR-CNNs from object detection to the bone age estimation problem, which enabled them to take the original x-ray images directly as inputs instead of first extracting manually the regions of interest from the x-ray images. The extracted features along with detected regions of interest are used to predict the bone age. In (48), the authors used DL to extract features and Extreme Gradient Boosting (XGBoost) algorithm to classify. They demonstrated that numerous sources of information can help in the rapid diagnosis of CPP without the requirement for a GnRH stimulation test. They revealed that the most important additional sources were LH levels and the uterine volume measured through pelvic US. Although pelvic US cannot be used alone to predict CPP in women, it improves the diagnosis when combined with laboratory data. As demonstrated in (37), the authors inferred a set of features that were useful for classification; however, they are not presently employed for CPP diagnosis.

More recently, in (33), the authors presented an artificial intelligence-based diagnostic system called dynamic multimodal variational autoencoder (DMVAE) to diagnose CPP. The datasets come from four different resources including electronic health records, laboratory tests, pelvic US, and left-hand radiography reports. All 2,228 subjects had electronic health records (with 10 features) and laboratory tests (with 9 features); however, only 858 subjects had left-hand radiography reports (with 6 features), and 896 subjects had pelvic US (with 16 features). The first step was to infer (impute) the missing scoring values for the subjects in cases of pelvic US and hand radiography reports, using variational auto-encoders (VAEs). VAE is a generative model consisting of an encoder and a decoder, which minimizes the error between the initial data and encoded–decoded data. Precisely, a modality indicator first specifies if the feature value is missing or not, and then the joint representation between different modalities is learned to impute the missing values. Next, a GBDT algorithm, known to perform well with high-dimensional datasets, was used to predict the response to a GnRH agonist stimulation given the combined features from all four resources. There were 1,046 positive stimulation tests and 1,182 negative stimulation tests. Finally, shapely additive explanations (SHAP) were employed to explain the output of the machine learning model at both the feature level and data source level. On the global feature scale, LH levels were the most important feature [as also shown by Pan et al. (48)] followed by the LH/FSH ratio. This is encouraging as it is consistent with the current clinical decision-making process. Laboratory tests were the most important sources followed by pelvic US reports. The least important source was the left-hand radiography reports. This work presents an excellent example of DL-based system that can help to resolve missingness problem in order to accurately predict if a patient needs to undergo GnRH stimulation test.

#### 3.1.4 Conclusion

BAA represents a perfect example of object detection where CNN-based DL models can perform efficiently. The task is to predict the bone age class given hand radiographs as inputs. The references described above show a true potential of DL for assessing bone age in clinical practice. In the future, we can expect that DL systems will be used routinely in clinical practice, just like feature extraction systems like BoneXpert (62) are. However, certain limitations have been raised in the literature and need to be addressed. BAAs are compared with the manual bone age determined by experts, yet one cannot confirm that the manually determined age is the true age. It would be wise to refer to the age assessed through other methods such as MRI (63). The bone maturity also differs across different ethnicities; one should encourage the use of datasets coming from multiple ethnicities as shown in the work of Kim et al. (36). In addition, DL models have an intrinsic black box nature. One can resolve this problem by interpreting model predictions. For example, in (37, 64), the authors highlighted the most sensitive image parts corresponding to specific anatomical zones. Larson et al. (64) discussed that the metacarpal–phalangeal joints, proximal interphalangeal joints, and carpal bones were the most sensitive areas, all of which correspond to maturity indicators as defined by GP criteria. More precisely, the sensitivity to carpal bone is similar to the TW method. Contrarily, it has been shown in (37) that although all features used by the TW method were kept, the carpal bones did not necessarily influence the final predictions, which raises the problem of the trustability of the proposed explanations. One can try using multiple methods to search for explanations and select the most pertinent for final analysis. In *Section 4.5*, we discuss several strategies to explain DL models. Finally, another frequently encountered problem is the reproducibility (see **Table 3**); either the source code is not available or the model is trained on private datasets (37). In the future, we can expect to see more advance DL methods that will be able to tackle the aforementioned issues.

### 3.2 Thyroid dysfunction

The thyroid gland is involved in many physiological functions, and is a major player in metabolism control, through the secretion of Triiodothyronine (T3, the active form) and Tetraiodothryronine (T4, converted into T3 by the target cells). As for the gonads, the thyroid activity is tightly controlled *via* endocrine loops within the thyreotropic axis. The perturbation of this endocrine dialogue can lead to either hypothyroidism or hyperthyroidism (see **Figure 1**). The most prevalent kind of thyroid problems is hypothyroidism (underactive thyroid), in which too little thyroid hormone is produced, and hyperthyroidism (overactive thyroid), in which too much thyroid hormone is generated.

Thyroid disorders are often difficult to diagnose based solely on clinical or laboratory investigations, as symptoms of hypothyroidism, such as weight gain, sadness, and exhaustion, are sometimes mistaken with other pathological conditions. Furthermore, other conditions such as pregnancy and psychiatric troubles might influence hormone levels, resulting in an incorrect diagnosis of thyroid dysfunction. The diagnosis of thyroid disorders also presents a challenging problem for machine learning algorithms, since there is typically a large variation between the numbers of samples belonging to different classes, i.e., hyperthyroidism (hyper), hypothyroidism (hypo), and normal (often the overrepresented status), resulting in an unbalanced dataset. Thyroid diseases are characterized as either functional (euthyroid or normal, hyper, and hypo) or structural (65). The structural categories are based on the morphology of the gland, which can be assessed by palpation (physical examination) or imaging techniques such as US or scintigraphy (which employs radioactive materials). The thyroid gland can be diffuse or nodular in structure, and if nodular, it can be mono-nodular or multi-nodular (66). Here, our main focus is the overview of functional classifications of the thyroid status using DL methods. The goal is to classify samples with different features from laboratory and routine tests into a hyper, hypo, or normal group. These features include serum total thyroxine (TT4), serum free thyroxine (FT4), triiodothyronine (T3), T3 uptake test (T3U), thyroxine binding globulin (TBG), serum thyroxine (T4), total serum triiodothyronine (T3 or T3RIA), T3 resin uptake (RT3U), serumthyroid-stimulating hormone (TSH), and increased TSH after injection of TSH-releasing hormone (ΔTSH), alkaline phosphatase (ALP), serum creatinine (S-Cr), low total cholesterol (T-Cho), mean corpuscular volume (MCV), and lactic acid dehydrogenase (LDH). It is also possible to use multiple sources of information such as images and medical records in addition to the laboratory tests as inputs to the ANN, which can help to improve the diagnosis. A synthetic overview on the DL approaches analyzed and compared in the next subsections is provided in **Tables 4**, **5**.

**Table 4:** DL-based studies for thyroid status assessment.

	Study	Method	Var	Dataset				Performance		Interpretation	Code
				Hyper	Hypo	Normal	Total	Accuracy	TPR		
Single Source	(67)	MLP, LVQ	6	49*	34	309	392	>93%	–	No	No
	(68)	MLP	21	166	368	6,666	7,200	>81%	–	No	No
Hybrid	(69)	MLP, RBF, and CSFNN	5	–	–	–	215	>79%	–	No	No
Multi source	(70)	MLP	23	–	–	–	176,727	–	>86%	Yes	Yes

The – sign implies that information was either missing or irrelevant for the corresponding study. ^∗^Thyrotoxic individuals instead of hyper cases were part of this study.

**Table 5:** Overview on the datasets and neural network architectures in a series of studies performed in Tohoku University.

Study	Train	Test	Vars	SOM	BRNN	Accuracy		Imbalance	Gender
				Competitive layer	Number of neurons	Hypo	Hyper		
(71)	215	–	5	30 × 30, *r* = 50	3, 5, 8, and 12	>83%	>91%	5× less	–
(18)	66	142	14	30 × 20, *r* = 30	8, 10, and 12	–	≈ 90%	∼2.6× less	♀/♂
(72)	120	171	14	30 × 20, *r* = 20	3–15	–	>81%	∼2.4× less	♀
(73)	78	135	14	30 × 20, *r* = 30	–	–	–	∼1.5× less	♂
(74)	156	307	12	30 × 20, *r* = 30	12	>80%	–	∼2.8× less	♀/♂

The imbalance column is used to indicate the degree of uneven data distribution. For example, in the first row, there were five times less samples from the minority class (hypo) than from the majority class (normal). The - sign implies that information was either missing or irrelevant for the corresponding study.

#### 3.2.1 Thyroid dysfunction classification—first application

The first exploratory work using ANNs to diagnose thyroid dysfunction dates back to 1993. Sharpe et al. (67) proposed a comparison of two types of ANNs, i.e., MLP and learning vector quantization (LVQ). LVQ is a supervised machine learning approach based on competitive learning in a similar manner as unsupervised SOM-based neural networks (see *Section 2.3*). The objective was to classify 392 cases with six features, T4, FT4, T3, T3U, TSH, and TBG, into three functional groups (hypothyroid, euthyroid, and thyrotoxic). The data were highly imbalanced, with 309 euthyroid (normal thyroid), 49 thyrotoxic, and 34 hypo cases. The authors pointed out the data imbalance issue (see Section 4.3) as well as that of pattern variations within classes. The training dataset should have enough pattern variation within a class to determine non-ambiguous decision boundaries within the feature space (75). To study the pattern variation issue, they used 30 examples in the training set encompassing the whole range of variations of six features for three groups. The results show a high classification rate of 96.4-99.7. It was unclear how they dealt with the data imbalance issue. Furthermore, the authors opted to divide their dataset into a training and a testing set, without building a validation test, because of the data scarcity. Nonetheless, their study forms a basis to explore ANN diagnostic systems for thyroid disorders.

#### 3.2.2 Data imbalance

Later, Zhang and Berardi (68) demonstrated the efficiency of ANNs to handle data imbalance issues, and the effect of sampling variability on the classification of thyroid status with a slightly bigger dataset of 7,200 cases. There were a total of 21 features representing a mixture of binary (15) and continuous (6) variables. The class distribution was again highly imbalanced with 5.1% cases belonging to the hypo group, 2.3% to the hyper group, and 92.6% to the normal group. A fourfold cross-validation scheme was deployed to ensure model robustness. The model was trained iteratively on three partitions of the dataset, and the fourth one was used for testing the performance, until all partitions had served as a testing set. To avoid any classification bias toward a particular class, it was made sure that there were enough examples from each class in each partition. They reported classification accuracy for each class separately. The average accuracy for the hyper, hypo, and normal group was 81%, 93%, and 99%, respectively, on the testing dataset. They also revealed that the basic logistic regression failed to deal with the imbalanced datasets and drastically overfitted the normal group; the average accuracy for the normal group was 100%, while it was 0% for the other two groups. Overall, a rather small variability in the classification rates was observed between training and testing examples of each class, except in the hyper group, suggesting that better strategies to cope with the imbalanced datasets are required. We discuss different strategies to deal with the data imbalance issue in *Section 4.3*.

#### 3.2.3 Hybrid network

In (69), the authors evaluated the use of three ANN architectures i.e., MLP, radial basis function (RBF), and adaptive conic section function neural network (CSFNN). RBF is a special type of two layer neural networks with a single hidden layer (11). The input layer of an RBF does not perform any computation; it simply forwards the input to the hidden layer just like a standard neural network. The role of the hidden layer is to transform the input space into a new, linearly separable, space. The number of nodes in the hidden layer is larger than the number of nodes in the input layer according to Cover’s theorem. This theorem states that, given a set of nonlinearly separable training examples, one can transform it into another linearly separable set, by casting it into a higher-dimensional space (76, 77). The hidden nodes of an MLP take the dot product between inputs and weights, and then apply an activation function (sigmoid, Tanh, Softmax, etc.) to compute the value of the node, while hidden nodes of RBF use the Euclidean distance between weights and inputs, and a Gaussian activation function (78). A CSFNN is a hybrid neural network where neurons behave either as MLP or as RBF, and as an intermediate unit. The dataset consists of 215 cases with five features. The objective was to learn the relationships between these features and three classes, i.e., hyper, hypo, and normal. The dataset was highly imbalanced as only 30 out of 215 cases belonged to the normal class. The ANN based on a hybrid structure (CSFNN) was the computationally most efficient architecture with a better accuracy than MLP and RBF. It was not clear whether the data imbalance problem was handled prior to training or not. The authors opted to divide their dataset into training and testing sets only. A threefold cross-validation scheme was deployed to ensure robustness.

#### 3.2.4 Predicting thyroid disorder on patient datasets

**Figure 10:**
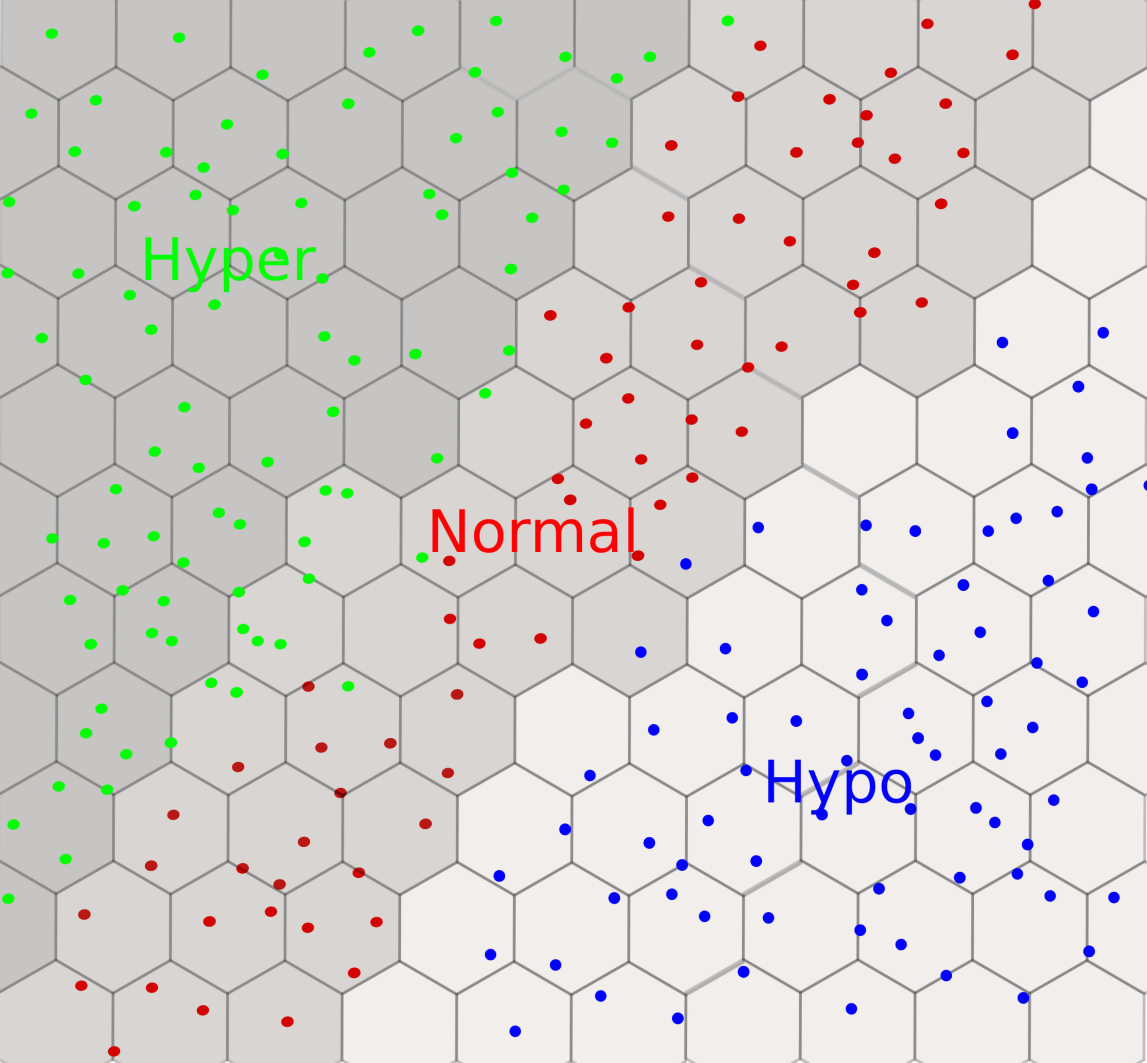
An example of a self-organizing map with a 2D hexagonal grid for thyroid dysfunction classification. We observe three clusters: hyper in green, hypo in blue, and normal in red. Each dot can be mapped to the input subject using the coordinates of the winner nodes on the grid.

In this section, we present in chronological order a series of studies undergone by a group of researchers from Tohoku University. A synoptic view of the designed neural networks is provided in **Table 4**. The first study, in 2005, was dedicated to the functional classification of thyroid status using two types of neural networks: (1) SOM-based neural networks (see Section 2.3) and (2) BRNN (see Section 2.4). They classified 215 subjects (with five features) into three groups: (1) 150 normal, (2) 35 hyper, and (3) 30 hypo. The features were obtained from laboratory tests: T4, T3, RT3U, TSH, and ΔTSH. Their results show three distinct clusters of hypo, hyper, and normal in the SOM visualization (see **Figure 10**). Within clusters, a further classification level was also observed. For instance, patients with severe hyper cases (high T4, T3RIA, and very low ΔTSH) were situated in the deeper zone, whereas patients with mild cases were placed on the boundary of the hyper and normal clusters. A few cases of hyper and hypo were wrongly labeled as normal. However, when the authors generated the SOM with only two variables, T4 and ΔTSH (identified by BRNNs as the most relevant ones), the classification accuracy improved. The authors argued that this could be because of the redundant role of RT3U for the hyper group and TSH for the hypo group. They also built a three-layer BRNN with and without the ARD method (see Section 2.4), and implemented a backward stepwise selection strategy to identify the relevant or essential features. In backward selection strategy, first a model is built with all variables and then variables having the least effect on the model’s performance are eliminated. They demonstrated that the best classifiers always used T4 and ΔTSH as inputs. However, the authors did not employ an independent testing dataset to validate their predictions (71). Later on, the authors used 14 features from only routine tests to classify patients into hyper and normal groups using a BRNN. They identified 3 relevant or important features, ALP, S-Cr, and T-Cho, out of 14. They also had a separate testing dataset, which was not the case in the previous study we discussed. It is interesting to note that three out of the seven individuals predicted to be in the hyper class by both SOM and BRNN were later diagnosed as such by a physician, and these three patients were in the deeper zone of hyper cases in the SOM. This points out the interest of using neural networks for thyroid dysfunction classification. Furthermore, the remaining four persons identified as hyper had hepatic dysfunction, which explains the incorrect classifications as routine test results from these patients mirrored the hyper instances (18). In a next study, they increased the sample size of the training and testing datasets (72). In addition, they generated 1,000 virtual subjects, by randomly generating values for different features using the mean and variance of the patient dataset, to verify the robustness of the screening method. The accuracy of the classification was improved with the augmented datasets, as participants with severe hyper cases were deeper in the hyper zone than in previous SOM visualizations. The false-negative rate was reported to be 10%, and was attributed to a comorbidity in addition to hyperthyroidism, which altered the routine test interpretation. For example, a patient with graves and a renal disease had a normal S-Cr level (an important variable for prediction with this BRNN). A strong association was also found between the three key variables (S-Cr, T-Cho, and ALP) and FT4.

Later on, the authors extended the work to men in order to account for sex differences in routine test data (73). In addition to two neural networks, they also build a model based on a support vector machine (SVM). We will not give details on SVMs as it is out of the scope of this paper. A same false-negative rate of 10% was reported as in the previous women-only study; however, the false-positive rate (six male subjects were predicted to be in hyper class) was higher due to more hepatic dysfunction cases in men than women. S-Cr, T-Cho, and ALP appeared to be the most important variables for diagnosis in men as in women. The authors did not make a detailed comparative study; they simply suggested that SVMs performed marginally better than BRNNs. After focusing on the detection of hyperthyroidism, the authors extended their approach to include hypo cases as well (74). Four variables, LDH, TC, S-Cr, and RBC, were the most important out of the 10 input variables. A strong correlation was reported between these four variables and TT4. A false-negative rate of 10% was reported again. False positives were often reported in elderly subjects, which the authors speculated may be due to a slow metabolism. Nonetheless, false positive was reported regardless of age if patients had additional conditions.

#### 3.2.5 Multiple sources of information

More recently, an explainable diagnostic support system was proposed in (70). A significantly larger dataset than aforementioned studies (18, 67–69, 71–74) of 176,727 subjects recruited in four hospitals was used in this study. The patients were labeled with 23 features (laboratory tests). Separate models were built to identify patients in hypo or hyper within the whole dataset, using four machine learning algorithms: GBDT, SVM, logistic regression, and MLP. For the hyper classification model, hypo and normal patients were used as a control group; for the hypo classification model, hyper and normal were used as a control group. We will not go into details of the first three machine learning algorithms as it is out of the scope of this paper. The GBDT model performed the best as compared to the other three models. S-Cr, MCV, and T-Cho were important variables for the hyper model, while S-Cr, LDH, and T-Cho were important features for the hypo model. A direct comparison cannot be performed with other studies, as lots of technical details are missing. Nonetheless, it is encouraging to observe that LDH, S-Cr, and T-Cho were the common variables identified as important features both in the previous works (72, 74) and in this study.

#### 3.2.6 Conclusion

Starting from 1993, there has been a huge amount of impressive studies based on DL methods to classify thyroid function, and their accuracy has improved with time. One surprising observation is the handling of imbalanced data. The imbalance issue arises from the unequal distribution of samples for each class, for example, more samples in the normal group than in the hyper or hypo group in a dataset. Most of the methods described above did not explicitly implement a strategy to handle this issue, and, still, they manage to achieve a high accuracy. However, sometimes (67), an overall accuracy metric was mentioned without specifying the accuracy for each class separately. Furthermore, the accuracy is not the best metric to measure the performance of a classifier in case of imbalanced datasets. A high global accuracy can be achieved even if the model fails to classify examples from the important class, i.e., hyper and hypo correctly, as demonstrated above (68) in case of the regression classifier. Furthermore, lots of important details were missing on dataset divisions, parameters, and architectures of DL models. It is valuable to have this information to perform fair comparisons and guarantee the reproducibility of results. Having said that, DL methods, when implemented correctly, can certainly help to improve the performance of diagnosis (79). For example, three out of seven subjects predicted as hyper by neural networks were later diagnosed so by a physician in (18). To go one step further, one can design a system that can take different types of medical exams (images and endocrine tests) as input and generate the diagnosis based on varying sources of information. Even with these necessary improvements, one should not assume that the model will cope with all scenarios; the presence of a diagnostician remains mandatory to confirm the predictions. Contrary to the statement made by an AI expert, “People should stop training radiologists now” (80), these systems will not replace the physicians in a foreseeable future, but will certainly serve as a second opinion.

## 4 Considerations

### 4.1 Preprocessing

We should perform some preprocessing before feeding our data into a model. One of the most prevalent issues is missing values. The missing data might be the result of a human or machine error. Because different types of variables, e.g., categorical versus numerical, require a distinct treatment, it is critical to apply datatype-specific manipulations. The severity of missing values depends on the percentage of missingness. The missingness problem can be resolved by either removing the examples with missing values (unpopular as we discard lots of information) or replacing missing values with an estimated value calculated through a technique known as imputation. One usually takes the mean, median, or mode to replace missing data (81). However, this approach can introduce bias in the dataset, especially if the percentage of missing values is substantial. For a detailed survey on how to tackle missing values, the interested reader can consult this overview (82). Missing values can also be predicted using machine learning methods. One needs to define a predictive model in order to extrapolate missing values from the available data. For example, in thyroid status classification (70), the k-nearest neighbor algorithm was employed to predict the missing values. To address the issue of missingness in a multi-source context, we can also deploy the “data-driven sparse Partial Least Square” method. This method imputes missing samples in covariate blocks in a supervised fashion to estimate the underlying model and to generate predictions (83). DL-based imputation methods are particularly useful for inferring missing data when there are complex, nonlinear relationships between features. VAEs can outperform the imputations obtained from using the mean or applying a principal component analysis (PCA) (84). In case of BAA (see Section 3.1.3), the authors in (33) have extended VAEs to multi-source settings (missing values in one source are predicted from other information sources), and improved both the classification and imputation performance.

After dealing with missing values, we have to convert the data into a machine-readable format. We choose the appropriate preprocessing method depending on the datatype, i.e., numerical, binary, or categorical. Although numerical data are already in a machine-readable format, we are faced most of the time with a situation where different features have variable ranges. To avoid the major artificial influence of differences in amplitude, we rescale the data on a same range, typically between 0 and 1. For binary variables, for example, gender, we can assign 1 to a female and -1 to a male, or *vice versa*. For categorical features, we can use integer encoding or one-hot encoding, among others (85). In integer encoding, each category is assigned a particular integer, for example, 1 to *k* for *k* categories. However, this introduces an ordinal relationship among categories, which might not be present originally. The other alternative is to use one-hot encoding, which converts each category into a binary vector of size *k* in case of *k* categories.

### 4.2 Data exploration

It is wise to perform a deep exploration of data before building a model. The initial exploration step helps to gather a basic understanding of the dataset and select the most robust algorithm for the task at hand. Different techniques can be used to identify patterns and interesting characteristics in the datasets. We can start with an unsupervised, linear data reduction method, i.e., PCA. We can also apply a nonlinear, unsupervised data reduction technique, i.e., t-distributed stochastic neighbor embedding (t-SNE) (86) for high-dimensional data exploration and visualization. The objective is to reduce the high-dimensional data into two or three dimensions where similar data points are close together. t-SNE defines the probability distribution of similar data points over the high and corresponding low-dimensional spaces, and then minimizes the distance between these probability distributions using a gradient descent algorithm. Alternatively, one can apply a uniform manifold approximation and projection (UMAP) method, which is faster and better at conserving the global structure than t-SNE (87).

### 4.3 Imbalanced data

Classification algorithms are known to be very sensitive to unbalanced data when the aim is to derive classification and prediction tools for categorical classes. In general, the algorithms will correctly classify the most frequent classes and lead to higher misclassification rates for the minority classes, which are often the most interesting ones. In the instance of thyroid status classification, we have much more normal examples (majority class) than hypo or hyper examples (minority class). Before building a classification model, we have to resolve the issue of imbalanced data by employing different techniques such as under- or oversampling of the majority or minority class, respectively.

We can perform undersampling with the edited nearest neighbor (ENN) algorithm (88). ENN starts with removing from the samples of the majority class whose class differs from that of their *k* nearest neighbors (*k* is typically an odd number to avoid ties). However, by performing undersampling, we can lose important information from the majority class. An alternative strategy is to perform a random oversampling of the minority classes to create a balanced dataset, but it may lead to overfitting the data. In order to overcome this issue, instead of simply copying examples, we can generate synthetic examples for the minority class using ADASYN (Adaptive Synthetic) (89) or SMOTE (Synthetic Minority Over-sampling Technique) (90) among others. SMOTE randomly selects examples from the minority class and creates a new synthetic data point between the selected example and one of its *k* nearest neighbors by interpolation, while ADASYN also takes into account the weighted distribution for minority class samples in order to create new examples. In the instance of thyroid status classification (see Section 3.2), we could deploy ADASYN or SMOTE to create synthetic samples for hypo or hyper groups. Furthermore, other methodologies such as ensemble modeling (91, 92) and different performance metrics [Cohen’s kappa (93) and Matthews correlation coefficient (94)] are available to tackle such issues. Practitioners should investigate which methods are suitable for their problem and how the application of these methods may impact the final results.

### 4.4 Hyperparameters

Hyperparameter tuning is needed to get the best-performing model. The hyperparameters (number of layers and neurons, learning rate) are different from the model parameters (weights), which are learned during training by optimizing the cost function (see **Figure 4** and equations 2 and 3). The hyperparameters are involved in the model design and are not updated during the training process (95). Hyperparameter tuning usually begins by constructing a model, then sampling values from a range of hyperparameter values, and finally assessing the model performance on the validation dataset. Remember that we separate our data into three categories: (1) training for learning model parameters, (2) validation for optimizing hyperparameters, and (3) testing for evaluating the generalizability of the model.

Different approaches are used to optimize the hyperparameter values. Each strategy has advantages and disadvantages. Manual search is a widely prevalent strategy, which uses a trial-and-error approach and requires expert knowledge (96). Random search (97) randomly selects a set of hyperparameters from a defined range of hyperparameters, as done in the grid search, instead of verifying each configuration exhaustively. In both the grid and random search, the search space is independent, so that parallelization is easy to achieve. Unfortunately, both methods ignore the results of earlier iterations. As a result, the algorithm may be stuck in unpromising areas of the search space, and it may take a long time to find optimal hyperparameters. Bayesian approaches (98), on the other hand, uses information from the previous iterations to set the hyperparameter values, so that they may need less time to tweak parameters by completing fewer iterations (99). A hyperband is a variant of the random search method based on pure-exploration principles. It implements an intelligent resource allocation as well as early stopping criteria. It randomly selects the configurations of parameters and discards the poorly performing ones using successive halving. It discards the worst configurations as early as possible because the most promising configurations frequently outperform the worst ones since the beginning. In terms of processing time, the hyperband algorithm outperforms the Bayesian (100) approach. However, in practice, it is usually difficult to verify all combinations of hyperparameters as we are mostly constrained by the computational resources. One can take guidance from hyperparameter optimization methods with a grain of salt. A detailed review on hyperparameter optimization methods can be found here (101).

### 4.5 The black box nature

It is often useful to understand the internal working of DL models and to identify the most essential features for the classification or regression tasks. This is a crucial precondition to get insight into the underlying biological or clinical structure of data and to ground any clinical translation. Some models are more explainable (white box models) such as linear models and decision trees. However, these models may not perform well in certain situations and we may need to go for more complex and powerful models such as DL models (black box models); i.e., the thought process behind a particular decision or prediction is not clear. DL models are successful in giving performances comparable to humans (102); however they are not a silver bullet for all kinds of problems. Sometimes, even for a difficult problem but with well-structured data, a simple model may perform likewise (103). That is why it is important to perform an initial data exploration to select the appropriate classification tool (see *Section 4.2*).

Fortunately, many methods have been developed in the last decade to tackle the problem of explainability of DL models, such as feature relevance, local or global explanations, and visualizations [for a review, see (103–106)]. Our aim here is to briefly describe different methods to turn the black box nature of DL models into a white box. Some of these methods are model agnostic and some are model specific (107). Model-agnostic methods do not depend on model design. A widely used agnostic method is LIME (Local Interpretable Model-agnostic Explanations), which explains the model by perturbing the dataset around the observation of interest (for example, an individual or an image) and analyzing how the model changes its predictions w.r.t. the perturbed dataset. An explanation for an observation is then generated by learning a simpler linear model using a subset of features. These explanations are locally truthful, i.e., only valid for the observation being investigated (108). A variant of LIME is Anchors (High-Precision Model-Agnostic Explanations), which explains the predictions by learning if–then rules, and reports the measure of precision associated with each explanation (109). For example, for the thyroid status classification (section 3.2), one can deploy LIME to score input features (e.g., levels of T3, FT4, and TBG). This will help to determine which features contribute to a hyer or hypo risk prediction and what are the relative weights using a linear approximation of the DL model. Saliency maps provide a model-specific way to explain CNNs by highlighting the important image fragments. The maps highlight regions of interest from an image or video by monitoring the changes in the output w.r.t. changes in the input image. They are obtained by repeatedly applying small modifications to an input image (110). Typically, saliency maps could be used to highlight the important image fragments of the left-hand radiographs in the framework of precocious puberty diagnostic (see Section 3.1). This would help one to see which anatomical zones of the left-hand radiographs influence the final prediction of an ANN model. In (35), the attention map of prepubertal category highlighted the mid-distal phalanges and carpal bones as important regions for classification. DeepLIFT (Deep Learning Important FeaTures) is a popular, heuristic-based, model-specific method. This method assigns contribution scores to the features of a particular observation, according to a reference point (neutral value). DeepLIFT basically tries to trace back contributions to the input features by back-propagating activated neurons (111). A choice of reference value for applying DeepLIFT requires careful consideration and domain-specific knowledge. For example, in case of thyroid dysfunction, we can select the clinical characteristics of a thyroid subject that did not experience any thyroid dysfunction as a starting point. DeepLIFT can be sensitive to the choice of the reference values. To achieve stable results, one can use DeepLIFT with multiple reference values. Despite the rapid development of these methods, the explainability of neural networks remains an open question.

## 5 Conclusion

DL is very effective in handling large amounts of data and finding patterns or functions hidden deep inside the biological datasets, where classical linear models may fail. With the availability of ready-to-use open-source DL libraries such as Torch, Keras, Caffe, Theano, MXNet, and DMTK, among others, it is becoming easier to apply DL out of the box without knowing much about the underlying theory. However, since DL application is a challenging task, one should not arbitrarily apply DL to any dataset. The successful application of DL on biological data requires a synergy of skills from mathematics, computer science, and biology, as well as navigation through different subtle points and caveats. One has to take into account the common issues arising from the data or chosen method, such as imbalanced data, parameter optimization, and the black box nature. Currently, there are many methods available to turn these black box neural networks into more white box models. However, the challenge remains of which explanation to trust, especially if different methods give contradictory explanations. One way to overcome this challenge would be to generate explanations from multiple methods, keeping only the most consistent ones. Another way would be to provide a confidence score with each explanation, and to trust only those explanations with a high score. Lastly, domain knowledge also plays an important role in the validation of these explanations. For example, we can add constraints, so that each explanation meets definite specifications (prior knowledge). Furthermore, at the beginning of DL application, as exemplified with precocious puberty and thyroid status classification, only one data modality was taken into account. In recent years, different sources of information are merged in order to develop more robust neural networks. Integrating different kinds of datasets, however, raises new issues, such as missingness, structural heterogeneity, datatype differences, and dynamic ranges. Further advances in DL methods are still awaited to handle these integration problems.

## Author contributions

MR, RY, and FC contributed to the conception and design of the study. MR collected and organized the material. MR wrote the first draft of the manuscript. All authors contributed to manuscript revision, read, and approved the submitted version.

## Conflict of interest

The authors declare that the research was conducted in the absence of any commercial or financial relationships that could be construed as a potential conflict of interest.

The handling editor is currently organizing a Research Topic with the author FC.

## Publisher’s note

All claims expressed in this article are solely those of the authors and do not necessarily represent those of their affiliated organizations, or those of the publisher, the editors and the reviewers. Any product that may be evaluated in this article, or claim that may be made by its manufacturer, is not guaranteed or endorsed by the publisher.

## Footnotes

1 We provide a list of abbreviations at the end of the paper.

## Glossary

**Table:**

DL	Deep learning
ANNs	Artificial neural networks
MLPs	Multilayer perceptrons
CNNs	Convolutional neural networks;
BRNNs	Bayesian regularized neural networks
ARD	Automatic relevance determination
SOMs	Self-organizing maps
FFNNs	Feed-forward neural networks
PP	Precocious puberty
GnRH	Gonadotropin-releasing hormone
PPP	Peripheral precocious puberty
CPP	Central precocious puberty
BAA	Bone age assessment
TW	Tanner–Whitehouse
GP	Greulich and Pyle
US	Ultrasonography
FR-CNNs	Faster region-based CNNs;
SHAP	Shapely additive explanations
T3	Triiodothyronine;
T4	Tetraiodothyronine or thyroxine
Hypo	Hypothyroidism;
Hyper	Hyperthyroidism
LVQ	Learning vector quantization;
RBF	Radial basis function
CSFNN	Adaptive conic section function neural network
GBDT	Gradient boosting decisiontree;
SVM	Support vector machines
t-SNE	t-distributed stochastic neighbor embedding
PCA	Principal component analysis
UMAP	Uniform manifold approximation and projection
ENN	Edited nearest neighbour
SMOTE	Synthetic minority over-sampling technique
ADASYN	Adaptive synthetic
LIME	Local interpretable model-agnostic explanations
Anchors	High-precision model-agnostic explanations
DeepLIFT	Deep Learning Important FeaTures;
XGBoost	Extreme gradient boosting
LDH	Lactic acid dehydrogenase
T-Cho	Total cholesterol
S-Cr	Serum creatinine
MCV	Mean corpuscular volume
TT4	Serum total thyroxine
ALP	Alkaline Posphatase
TSH	Thyroid-stimulating Hormone
T3U	T3 Uptake
FT4	Serum Free Thyroxine
ELM	Extreme Learning Machine
TBG	Thyroxine Binding Globulin
RT3U	T3 Resin Uptake
∆TSH	Increased TSH after injection of TSH-releasing hormone
VAE	Variational Auto-encoders
